# Antimicrobial Effects of AH26 Sealer/Antibiotic Combinations Against *Enterococcus Faecalis*

**Published:** 2008-10-01

**Authors:** Hasan Razmi, Kazem Ashofteh Yazdi, Fereshteh Jabalameli, Shaghayegh Parvizi

**Affiliations:** 1 *Department of Endodontics, Dental School, Dental Research Center, Tehran University of Medical Sciences, Tehran, Iran*; 2 *Department of Microbiology, School of Medicine, Tehran University of Medical Sciences, Tehran, Iran*; 3 *Department of Endodontics, Dental School, Tehran University of Medical Sciences, Tehran, Iran*

**Keywords:** AH26, Amoxicillin, Antimicrobial, Doxycycline, Endodontics, Sealer

## Abstract

**INTRODUCTION:** The purpose of this *in vitro* study was to evaluate the antimicrobial effects of two antibiotics added to AH_26_ sealer against *Enterococcus faecalis* (EF).

**MATERIALS AND METHODS:** The antimicrobial effects of two antibiotic (amoxicillin and doxycycline) that were added separately to AH_26_ sealer was evaluated by using the agar diffusion test and *in vitro* human root inoculation method. The freshly mixed sealers were placed in prepared wells inside agar plates inoculated with EF, and were incubated at 37^°^C. The zones of inhibition were measured at 24, 48, 72 hours and 7 days. Root specimens were prepared and obturated with lateral condensation technique. Samples were collected from infected root canals after 2 and 7 days of incubation and the number of colony-forming units (CFU) was determined. The data were analyzed using one-way and two-way ANOVA.

**RESULTS:** The findings of this study revealed that sealer-antibiotic combination containing amoxicillin and doxycycline had a significant difference in the mean zones of inhibition when compared to AH_26_ sealer alone in all of the time periods (P<0.05). The minimum effective concentration against EF was one percent of sealer powder weight for both of amoxicillin and doxycyline. The mean log_10 _CFU in AH_26_-doxycyline combination group was significantly lower than other groups at 48 h incubation period (P<0.05). AH_26_-doxycycline combination group and AH_26_-Amoxicillin combination group killed bacteria (mean CFU=0) in the dentinal tubules at 7 days incubation period.

**CONCLUSION:** *In vitro* root fillings carried out with gutta-percha and AH_26_-antibiotic combination were effective in killing EF in dentinal tubules.

## INTRODUCTION

Elimination of microorganisms from the root canal system is the one of the most important objectives of RCT (1). *Enterococcus faecalis* (EF) has been the most frequently identified species in canals of root-filled teeth with periapical lesions. EF is extremely resistant to current treatment modalities in endodontics. Therefore, the success rate of root canal treatment can be significantly reduced with the presence of this microorganism at the time of obturation (2,3). Sealers having antimicrobial effects may be able to overcome some of the limitations of chemomechanical preparation (4).

Within the last few years, antibiotics have been used in dentistry systemically and topically. Chronic periradicular lesions associated with pulp necrosis do not have adequate blood supply. So, the concentration of antibiotics reaching root canal system in systemic administration is negligible and not beneficial (5). Systemically administered antibiotics have some complications such as toxicity, allergic reaction and development of resistant strains of microorganisms (6). It has been reported that the main advantage of local antibiotics compared to systemic use is that systemic complications are prevented and that substantially higher concentrations can be used (7).

Susceptibility of EF to various antibiotics, such as amoxicillin, vancomycin, erythromycin, benzyl penicillin and doxycycline has been assessed in a series of studies (8-10).

AH_26_ is an epoxy resin that initially was developed as a single-filler material. Because of its positive handling characteristics, it has been extensively used as a sealer. It has a good flow, adapts well to dentin walls and allows for sufficient working time (11). Like most sealers, AH_26_ is very toxic when freshly prepared. However, this toxicity declines rapidly during setting; and after 24h, the cement has one of the lowest toxicities of endodontic sealers (12).

To date, limited studies have been performed to assess the incorporation of antibiotics to endodontic sealers and only one study (13) has evaluated the antimicrobial effects of five antibiotics when individually added to Kerr EWT sealer against EF, using Agar Diffusion Test (ADT).

The purpose of this *in vitro* study was to evaluate the antimicrobial effects of amoxicillin and doxycycline when added individually to AH_26_ sealer against EF, using agar diffusion test and also in experimentally infected dentinal tubules with this microorganism.

## MATERIALS AND METHODS

*Enterococcus faecalis* (*EF*, ATCC 29212) was obtained from the American Type Culture Collection (Manassas, VA). The bacterium was grown and maintained on Brain Heart Infusion (BHI) agar or broth (Difco, Sparks, MD, USA). To preserve the bacterium and its characteristics, upon receipt, cultures were frozen (-20^º^C) in vials with glycerol from which new stock cultures were periodically established. A culture of EF was grown overnight at 37^º^C in BHI broth. Bacterial growth was checked by changes in turbidity at 24 hours. BHI agar plates were inoculated with EF by spreading the culture over the surface of the plate with a cotton swab to develop a lawn of cells. The bacteria were cultivated in solid media, and broth culture suspensions were prepared and adjusted to No.1 Mcfarland standard (3×10^8^ cells/mL) (14).

Aliquots of the suspension containing EF were spread on four 140-mm diameter Petri dishes containing Mueller-Hinton Agar medium (Merck, Darmstadt, Germany). Excess inoculum was removed with a pipette, and the inoculated plates were dried for 15 minutes at 37^°^C. Each plate was divided into 7 sections for placement of AH_26_-antibiotic combination in six concentration (0%, 1%, 5%, 10%, 25% and 50% of the powder of AH_26_ sealer) as well as distilled water as the control. In each section of the plate, a well 5 mm in diameter was created with a sterile stainless steel cylinder.

Two antibiotics: amoxicillin (Iran Daru, Iran) and doxycycline (Iran Daru, Iran) were prepared with a mortar and pestal (13) and were added separately to the powder of AH_26_ sealer (Dentsply, DeTrey, Konstanz, Germany) in each concentration and mixed according to manufacturers’ specifications. A sample of the freshly mixed dental material was placed into wells of each section. Each experiment was replicated 4 times. All plates were incubated for 7 days at 37^º^C under aerobic condition, and zones of growth inhibition were measured at 24, 48, 72 hours and 7 days. The data were analyzed using one-way and two-way ANOVA and multiple comparisons were made by Tukey tests. P-values less than 0.05 were considered statistically significant.

Eighty-four recently extracted single-rooted human teeth that were approximately of the same dimensions were selected and stored in saline solution, until required. The crowns of the teeth were sectioned at CEJ and all the roots were adjusted to 13 mm working length. Patency of the apical foramen was determined with a size 10 or 15 k-file (Dentsply, Maillefer, Tulsa, OK, USA). The working length was established 1 mm short of the apical foramen. After coronal flaring with #3 and #4 Gates-Glidden drills (Dentsply, Maillefer, Tulsa, OK, USA), instrumentation was completed with Rotary profile files (Dentsply-Tulsa Dental, Tulsa, OK, USA) in a crown-down technique. The size 35 with 0.06 taper profile was taken to working length in each of the canals. Canals were irrigated with saline throughout the instrumentation. The canals were also irrigated with 1 mL of 17% EDTA and 1 mL of 5.25 NaOCl to remove the smear layer. Finally, root canals were flushed with saline solution and dried with paper points. All of specimens were flushed with saline solution and dried with paper points. They were then mounted in resin blocks and sterilized by autoclaving for 20 min at 121^0^C. Root specimens were subsequently infected by using EF ATCC 29212 and then were kept in 50 mL of tryptic soy broth at 37^º^C for 4 weeks, during which time the broth was changed at 3-day intervals.

**Table 1:** Mean (SD) diameter of the of inhibition zones with AH_26_-Amoxicillin combination

Material	Duration of incubation			
	24h	48h	72h	7 days
**AH**_26_ ** sealer**	14/6(0/5)	14/3(0/5)	14/0(0/5)	14/0(0/5)
**AH**_26 _ **/ 1% **	35/6(0/5)	35/0(1/0)	34/0(0/0)	33/3(0/5)
**AH**_26 _ **/ 5% **	34/6(1/0)	34/6(0/5)	34/3(0/5)	33/3(0/5)
**AH**_26 _ **/10% **	34/6(0/5)	34/3(0/5)	33/3(0/5)	32/3(0/5)
**AH**_26 _ **/25 % **	36/3(1/5)	35/6(1/5)	34/6(1/5)	33/6(1/5)
**AH**_26 _ **/50 % **	36/3(0/5)	36/3(1/1)	35/2(0/5)	34/0(1/0)

The samples were randomly divided into the following treatment groups:

***1)*** negative control (n=6): the samples were sterilized without bacterial colonization and obturation,

***2)*** positive control (n=6): the samples were colonized with EF without obturation,

***3)*** (n=12): the root canals were filled with gutta-percha (Ariadent, Tehran, Iran) and AH26 sealer (Dentsply, De Trey, Konstanz, Germany) using cold lateral condensation technique,

***4)*** (n=12): The root canals were filled with gutta-percha and AH26-amoxicillin combination (the amount amoxicillin was 1% of the powder of AH26 sealer) using cold lateral condensation technique, and ***5)*** (n=12): the root canals were filled with gutta-percha and AH26-doxycycline combination (the amount of doxycycline was 1% of the powder of AH26 sealer) using cold lateral condensation technique.

All samples were incubated at 37^º^C and evaluated after 48 h and 7 days. On completion of incubation, all root canals were reestablished with sterile #3 and #4 Gates-Glidden drills and Hedstrom files. The dentin powder of 1-mm inner surface of the root canal was removed with new sterile Hedstrom files.

The dentin powder obtained from each specimen was immediately collected in sterile petri dishes. The samples were then transferred to test tubes containing 2 mL of phosphate buffered saline (PBS; Bio Whittaker, Verviers, Belgium), vortexed for 10s and diluted to a concentration of 10^-4^. Portion of 25 µL were inoculated onto TSB agar with 2 mgmL^-1^ streptomycin. Following incubation for 48h and 7days at 37^º^C, visible colonies were counted and the total Colony Forming Units (CFU) calculated. When growth occurred, the bacteria were subcultured on TSB agar plates and checked for purity and identity of EF.

**Table 2:** Mean (SD) diameter of the inhibition zones with AH_26_-Doxycycline Combination

Material	Duration of incubation			
	24h	48h	72h	7 days
**AH**_26_ ** sealer**	14/6(0/5)	14/3(0/5)	14/0(1/0)	14/0(0/5)
**AH**_26 _ **/ 1% **	20/3(0/5)	19/6(0/5)	19/5(0/5)	19/3(0/0)
**AH**_26 _ **/ 5% **	20/2(1/0)	19/4(0/5)	19/3(0/5)	19/3(0/5)
**AH**_26 _ **/10% **	20/4(0/5)	19/3(0/5)	19/3(0/5)	19/3(0/5)
**AH**_26 _ **/25 % **	20/ 6(0/5)	19/2(0/5)	19/ 0(0/0)	19/0(0/5)
**AH**_26 _ **/50 % **	21/4(0/5)	19/6(0/5)	19/3(0/5)	19/0(1/0)

The CFU values were transformed to their log_10_ values and analyzed by two-way ANOVA. Significance was set at the 5% level.

## RESULTS

**ADT: **The mean diameters of inhibition zones caused by AH_26_-antibiotic combination are presented in Table 1 and Table 2.

“AH26 sealer” and “AH26 sealer and antibiotic combinations” containing amoxicillin and doxycycline caused inhibition zones in all of the concentrations. However, these combinations had a significant difference in the mean zones of inhibition when compared to AH26 sealer alone (P<0.05).

Result revealed that all sealer/antibiotic groups exhibited antimicrobial activity peaking around 1% concentration of antibiotic. This concentration was the minimal effective concentration and significant difference was found between this and concentrations of 5%, 10%, 25% and 50% antibiotics (P<0.05).

The data analysis revealed that AH_26_/ amoxicillin combination had significant larger inhibitory zones than AH_26_/doxycycline combination in all concentrations (P<0.05).

**Root dentin specimens: **The mean Log_10_ CFU for the various test groups are presented in Table 3. Bacteria were found in all positive control samples. (Log_10_ CFU=9.60) and counts were zero in all negative control samples (Log_10_ CFU=0).

**Table 3:** Mean log_10_ of the number of CFU in root dentin Collected from test specimen

Material	Duration	
	48 h	7 days
AH_26_ sealer	4.90	3.92
AH_26_ Amoxi	4.68	0
AH_26_ Doxy	4.06	0

**Table 4:** Mean, Standard deviation and standard error of the number of CFU in dentin Chips after 48 hours

Material	Mean		Standard deviation	Standard error
AH_26_ sealer (n=12)		8.000.000	101264.72	29232.60
AH_26_/Amox (n=12)		48333.333	55568.68	16041.29
AH_26_/Doxy (n=12)		46666.667	18006.73	5195.09

The mean Log_10_ CFU in all test groups was significantly lower than in positive control group (P<0.05). The mean Log_10_ CFU for AH_26_-doxycycline combination (4/06) was lower than that of AH_26_-amoxicillin combination (4.68) and AH_26_ sealer (4.90) at 48 hours (Table 4) but only the difference between AH_26_-doxycyclin combination and AH_26_ sealer was statistically significant (P<0.05).

AH_26_-amoxicillin and AH_26_-doxycycline combination totally killed bacteria (mean CFU=0) in the dentinal tubules after application for 7 days (Table 5).

The mean CFU for AH_26_-amoxicillin and AH_26_-doxycycline combination at 7 days was significantly lower than the mean CFU in AH26 sealer group at 48 hours (P<0.05). But this difference was not significant with AH_26_ sealer (P>0.05).

## DISCUSSION

The persistence of microorganisms in the root canal system often leads to failure of root canal treatment. EF was chosen because it is associated with persistent apical inflammation in clinical situations and may be difficult to eliminate from root canal system (15,16). Furthermore, EF is able to survive the antimicrobial effect of calcium hydroxide (CH) and sodium hypochlorite (NaOCl). "The survival of EF in CH appears to be unrelated to stress induced protein synthesis, but more the result of a functioning proton pump that drives protons into the cell to acidify the cytoplasm. The survival in NaOCl has been attributed to the possibility that the solution is buffered by dentin or variation in some EF strain that may allow them to survive low concentration of NaOCl" (16,20).

**Table 5:** Mean, Standard deviation and standard error of the number of CFU in dentin Chips after 7 days

Material	Mean	Standard deviation	Standard error
AH_26_ sealer (n=12)	8.500.000	10915.04	3150.90
AH_26_/Amox (n=12)	0	0	0
AH_26_/Doxy (n=12)	0	0	0

Antimicrobial agents are in two forms: antiseptics are very effective antimicrobial agents but they also tend to kill mammalian cells at similar concentrations that kill microorganisms. This toxicity is time dependent so their use should be limited to short term contact. Furthermore, antibiotics are less toxic to mammalian cells at effective concentrations and they are not suitable for short-term use (5).

The current study showed an increase in antimicrobial activity against EF when amoxicillin and doxycycline are added separately to AH_26_ sealer. Numerous studies have evaluated the susceptibility of EF to antibiotics (8-10,13). Two other studies reported a higher susceptibility of EF to amoxicillin, amoxicillin-clavulanic acid, benzylpenicillin, vancomycin and doxycyline with decreased susceptibility to erythromycin and azithromycin (9,10). Also, high susceptibility of EF to amoxicillin, doxycycline, penicillin and clindamycin but not to metronidazole has been demonstrated (13).

In contrast, one study demonstrated that EF was resistant to benzylpenicillin, ampicillin, clindamycin, metronidazole and tetracycline but sensitive to erythromycin and vancomycin (8). These differences may be related to the EF strain, treatment methods and origin of the endodontic infection.

AH_26_ was chosen as the test sealer because of it’s easy handling characteristics, good flow, good sealing to dentin , sufficient working time (12), prominent antimicrobial activity (21,22) and optimal depth of penetration into dentinal tubules in clinical situations (23).

Root canal sealers have limited and variable dentinal tubule penetrations The depth of penetration of canal sealer EWT into dentinal tubules was <100µ in several studies, one of the poorest (23,26). On the other hand, the mean maximum penetration depth in decreasing order was AH_26_ (1337µ), EndoREZ (863µ) and AH-plus (54.6µ) (23,26).

Bodrumlu *et al.* reported that the mean diameter of zone of inhibition caused by AH_26_ sealer, 14.8 mm, 14.3 mm, 13.8 mm at 24 hours, 48 hours and 72 hours respectively (27). In the current study, a similar mean zone of inhibition was seen with AH_26_ sealer in comparable time periods (14.6 mm, 14.3 mm, 14 mm respectively).

The results of this study revealed that all sealerantibiotic groups exhibited antimicrobial activity peaking around 1% concentration of antibiotics but Hoelscher *et al.* reported that the amount of antibiotic added to Kerr EWT sealer for gaining maximum antimicrobial activity was 10% (13).

Furthermore, in current study and Hoelscher’s study (13), the mean diameter of the growth inhibition zones of amoxicillin in sealer-antibiotic combination was significantly larger than sealer-doxycycline combination (P<0.05).

Haapasalo and Ørstavik (16) suggested that the presence of cementum affected the ability of EF cells to infect the dental tubules but another study reported that the penetration of EF, with or without cementum removal was similar (28). Therefore, it was decided not to remove the cementum in this study.

The choice of 4 weeks incubation period in this study was based on result of experiments that showed that EF did not invade the dentinal tubules until after 2 weeks of incubation, after 3 weeks of incubation a dense tubule infection occurred, and thereafter the depth of tubule infection only increased slowly over time (16).

Several studies reported different results about the penetration of EF within dental tubules such as 50 to 100µ (17), 270µ (29), and 300 to 400µ (16).

Therefore, in current study, dental shaving was collected from 1-mm (1000µ) inner surface of root canal.

The results of our study revealed that AH_26_-amoxicillin and AH_26_-doxycyline combination groups killed all of bacteria in the dental tubules after 7 days incubation period but AH_26_ sealer group did not. This condition may be related to formaldehyde release with prominent antibacterial effects in this sealer. When mixed, the hardener in this material, hexamethylene-tetramine, releases formaldehyde in an amount increasings over the 2-day setting period. Once set, the formaldehyde concentration in ~×200 that of the fresh mix and subsequently decreases over the next 7 days (30).

The present study found that certain antibiotics, when added to AH_26_ sealer, increased the antibacterial activity of the sealer against EF. Future research are needed to investing how the addition of an antibiotic may affect the sealing capacity, cytotoxic properties, physico-mechanical properties and *etc.* of an endodontic sealer.

However, it is necessary to explain that physicomechanical properties (setting time, working time, flow rate, solubility, film thickness and dimensional changes after setting) and the amount of antibiotic release have been evaluated by researchers of this study.

## CONCLUSION

Amoxicillin and doxycycline, when added to AH_26_ sealer, enhanced the antibacterial activity of the sealer against EF and the use of these sealers with gutta-percha in root filling *in vitro* was effective in killing EF in experimentally infected dentinal tubules.
